# Targeted Therapies in Oral and Oropharyngeal Cancer: An Overview of Emerging and Repurposed Agents

**DOI:** 10.3390/cancers17233761

**Published:** 2025-11-25

**Authors:** Geetpriya Kaur, Neetu Sinha, Nuno Vale, Rui Amaral Mendes

**Affiliations:** 1 PerMed Research Group, RISE-Health, Faculty of Medicine, University of Porto, Alameda Professor Hernâni Monteiro, 4200-319 Porto, Portugal; up202314194@edu.med.up.pt (G.K.); up202401667@edu.med.up.pt (N.S.); 2 RISE-Health, Department of Community Medicine, Health Information and Decision (MEDCIDS), Faculty of Medicine, University of Porto, Rua Doutor Plácido da Costa, 4200-450 Porto, Portugal; 3 Laboratory of Personalized Medicine, Department of Community Medicine, Health Information and Decision (MEDCIDS), Faculty of Medicine, University of Porto, Rua Doutor Plácido da Costa, 4200-450 Porto, Portugal; 4 Department of Community Medicine, Health Information and Decision (MEDCIDS), Faculty of Medicine, University of Porto, Rua Doutor Plácido da Costa, 4200-450 Porto, Portugal; 5 Department of Oral and Maxillofacial Medicine and Diagnostic Sciences, Case Western Reserve University, 10900 Euclid Ave., Cleveland, OH 44106-7342, USA

**Keywords:** oral cancer, oropharyngeal cancer, targeted therapy, drug repurposing, EGFR inhibitors, PD-1 blockade, metformin, statins, NSAIDs, PIK3CA

## Abstract

Oral and oropharyngeal squamous cell carcinomas (OSCC and OPSCC), two major sub-types of Head and Neck cancer, remain associated with significant morbidity and exhibit poor prognosis, with limited response to conventional therapies in advanced stages. Recent therapeutic strategies have increasingly focused on molecular targets involved in tumor proliferation, angiogenesis, and immune evasion. This overview provides a concise synthesis of targeted therapies under investigation or already in clinical use, including monoclonal antibodies against epidermal growth factor receptor (EGFR) (e.g., cetuximab) and immune checkpoint inhibitors (e.g., nivolumab, pembrolizumab), as well as inhibitors of programmed cell death protein 1 (PD-1) and its ligand (PD-L1) or agents targeting angiogenic and intracellular signaling pathways such as VEGF and mTOR. Alongside these novel agents, growing interest surrounds the repurposing of established pharmacological agents which appear to modulate tumor-related inflammation, metabolic dysregulation, and epithelial-to-mesenchymal transition. Metformin and statins, for instance, have demonstrated anti-proliferative and pro-apoptotic effects in preclinical OSCC models. Notably, recent evidence suggests that regular use of nonsteroidal anti-inflammatory drugs (NSAIDs), including aspirin, may improve survival specifically in patients with PIK3CA-altered Head and Neck tumors, potentially through modulation of the COX-2/PGE2 axis. Although prospective evidence remains limited and somewhat heterogeneous, existing preclinical and observational studies suggest that these agents may improve survival and reduce treatment-related toxicity, further pointing to the relevance of molecular stratification in guiding future repurposing strategies. This article aims to map the current therapeutic landscape, highlighting both established molecular targets and emerging repositioned drugs in the management of OSCC and OPSCC.

## Simple Summary

Oral and oropharyngeal cancers are aggressive and often respond poorly to standard treatments. This article explores targeted therapies that aim at specific molecules involved in tumor growth and immune evasion. It also highlights the potential of repurposing common drugs, like metformin, statins, and aspirin, which may slow tumor progression and reduce side effects. These drugs could be especially useful for patients with certain genetic tumor profiles. By reviewing both current and emerging therapies, this research aims to guide future studies and help develop more effective, personalized treatment options for patients with these challenging forms of cancer.

## 1. Introduction

According to the Global Cancer Observatory (GLOBOCAN) 2022 estimates, oral cancer ranks as the sixth-most common cancer worldwide, accounting for approximately 389,846 new cases and 188,438 deaths annually. South and Southeast Asia have the highest incidence of oral cancer, with oral cancer comprising up to 40% of all malignancies in countries such as Bangladesh, India, and Sri Lanka. In India, it is the most frequent cancer in men, accounting for nearly 30% of all cases [1].

Oral squamous cell carcinoma (OSCC) and oropharyngeal squamous cell carcinoma (OPSCC) are among the most common Head and Neck cancers (HNCs), a heterogeneous group marked by substantial variability in anatomy, histology, and pathophysiology. Risk factors include tobacco smoking and chewing, alcohol consumption, chronic infections with Candida albicans, HPV, EBV, as well as chronic inflammation, all of which have been found to contribute to the initiation and progression of oral cancers. Given the prognostic differences between HPV-positive and HPV-negative cases, NCCN guidelines have since included HPV status into therapeutic decision-making [2,3].

Surgical treatment and radiotherapy can lead to temporary or permanent impairment of speech, swallowing, and oral intake; changes in physical appearance may further contribute to social isolation. Notably, suicide rates among patients with Head and Neck cancer are approximately double those observed across other malignancies. Until recently, advances were largely incremental, and for many patients, standard treatment changed little over more than a decade [4].

Survivorship often entails lifelong attention to psychosocial well-being alongside medical care. The diagnosis presents many hardships, and a substantial proportion of patients struggle with daily challenges and uncertainty about how to move forward. Long-term care needs are complex and typically require a multidisciplinary team, with medical and radiation oncologists, oral and plastic surgeons, oral medicine specialists, rehabilitation professionals, speech and swallowing therapists, and nutritionists, working together to restore basic functions or prevent their deterioration [5,6].

Recent studies have significantly improved the conventional understanding of OSCC/OPSCC pathogenesis by identifying key molecular processes that drive carcinogenesis. It has been demonstrated that epigenetic modifications, genetic abnormalities, and alterations in cell signaling pathways, specifically those involving the p53 tumor suppressor gene, epidermal growth factor receptor (EGFR), and the PI3K/AKT/mTOR axis, drive tumor development and progression [7,8].

Thus, therapeutically, conventional surgical management, radiation therapy, and chemotherapy protocols continue to evolve with better outcomes and lesser side effects. However, most importantly, targeted and immune-based approaches, and repurposing of certain drugs, are undergoing clinical trials, with gene therapies and personalized treatments on the horizon. Drugs against defined drivers (e.g., EGFR) offer new options for patients with advanced or recurrent OSCC, while immune checkpoint inhibitors (ICIs), such as PD-1/PD-L1 inhibitors, have shown encouraging clinical activity by reinvigorating anti-tumor immune responses. This narrative review aims to provide a comprehensive overview regarding recent advances in targeted therapies and repurposed agents for OSCC and OPSCC, emphasizing the mechanistic rationale and translational relevance.

## 2. The Biological Mechanisms of Head and Neck Carcinogenesis

Genetic mutations, epigenetic modifications, and environmental variables interact intricately to cause oral and oropharyngeal cancer, a multi-step process starting as a premalignant lesion, like erythroplakia or leukoplakia, and evolving into invasive carcinoma. The pathophysiology includes molecular mechanisms that lead to accumulation of mutations along various signaling pathways, controlling immune evasion, apoptosis, differentiation, angiogenesis, and cellular proliferation. Developing tailored medicines and enhancing patient outcomes requires an understanding of these pathways [9].

### 2.1. Cell Signaling Pathways and Oncogenes

**Figure 1:**
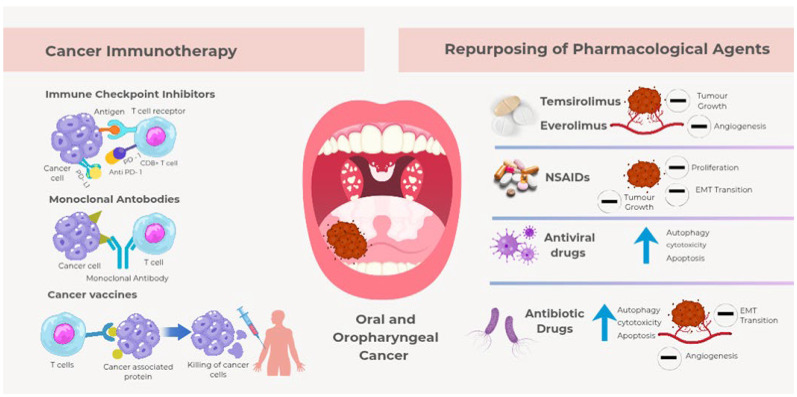
Targeted therapies and drug repurposing in oral and oropharyngeal cancer (Source: Originally developed using Canva).

The main signaling pathways that have been described in Oral and Oropharyngeal cancer (Figure 1) include the AS-RAF- MEK-ERK and PI3K/AKT/mTOR pathways, responsible for cell survival and proliferation; WNT/beta catenin signaling pathway; JAK-STAT signaling pathway; NOTCH signaling pathway; HIF-VEGF signaling pathway; and HIPPO signaling pathway. Moreover, several oncogenes that cause malignant transformation through dysregulated cell signaling pathways are activated in oral cancer. Within OSCC, one of the most commonly overexpressed oncogenes is the epidermal growth factor receptor (EGFR). When its ligands activate the transmembrane receptor EGFR, it activates downstream signaling cascades, such as the PI3K/AKT/mTOR and MAPK/ERK pathways, which facilitate cell invasion, survival, and proliferation. Since EGFR overexpression has been linked to poor prognosis and resistance to traditional treatments, monoclonal antibodies such as cetuximab target it.

In oral cancer, mutations in genes such as PIK3CA and PTEN often cause dysregulation of the PI3K/AKT/mTOR system. Hyperactivation of this system, which supports cell growth, survival, and metabolism, adds to the aggressive nature of OSCC. One possible therapeutic strategy for treating oral cancer is to target this system using certain inhibitors [10,11].

### 2.2. Tumor Suppressor Genes Mutations

One of the hallmarks of oral cancer development is inactivation of tumor suppressor genes. Among these, TP53, which codes for the p53 protein, is essential for controlling apoptosis, DNA repair, and the cell cycle, and is found to be mutated in 50–80% of cases. Mutations ranges from deletions, insertions, frameshift mutations, and point mutations, increasing the risk of malignant transformation and unfavorable progression of the tumors. Missense mutations in TP53 are associated with metastasis and poor prognosis. After TP53, CCND1 gene overexpression is observed in 30–46% of cases, resulting in unchecked proliferation of mutated cells and leading to lymph node metastasis, recurrences, and lower survival rates [12,13].

The p16 protein, a crucial cell cycle regulator, is encoded by CDKN2A, another essential tumor suppressor gene that is the third-most commonly inactivated gene in oral cancer. A vital checkpoint is eliminated when p16 function is lost, either by genetic mutations or epigenetic silencing, allowing unchecked cellular proliferation. Since p16 overexpression is frequently employed as a biomarker for HPV-driven carcinogenesis, this is especially pertinent to HPV-associated oral malignancies [14,15]. CDKN2A is frequently inactivated, either via deletion (more common in HPV-negative OSCC) or promoter hypermethylation, with reported associations to adverse pathological features; however, its prognostic impact appears context-dependent across HPV status and anatomical subsites [16].

### 2.3. Tumor Microenvironment (TME) and Immune Evasion

In oral cancer, the tumor microenvironment (TME) interacts dynamically with malignant cells to promote growth, invasion, metastasis, and resistance to radiotherapy/chemotherapy. The TME comprises the extracellular matrix (ECM), immune cells (including tumor-associated macrophages and myeloid-derived suppressor cells), endothelial cells, and cancer-associated fibroblasts (CAFs). Variation in the type and density of immune infiltrates influences not only treatment response and effectiveness but also tumor progression and prognosis [17].

Immune evasion is a defining feature of the TME in OSCC/OPSCC. Tumors upregulate immune checkpoint ligands, most notably PD-L1, which binds PD-1 on T cells and suppresses their activation. Immune checkpoint inhibitors (ICIs) such as pembrolizumab and nivolumab can restore anti-tumor immunity by blocking the PD-1/PD-L1 axis. Additional biomarkers (e.g., CTLA-4, PD-L1 combined positive score, tumor mutational burden, and measures of tumor-infiltrating lymphocytes) are increasingly used to guide ICI eligibility or to stratify risk. Elevated IL-6/STAT3 signaling and PI3K pathway activation (including PTEN loss) correlate with immunosuppressive myeloid infiltration and CD8^+^ T-cell exclusion, features linked to primary resistance to PD-1 blockade [18,19,20].

Beyond immune escape, the TME facilitates invasion and metastasis through secretion of matrix metalloproteinases (MMPs), which remodel the ECM and enable cellular migration. Hypoxia within the tumor core further drives angiogenesis and therapy resistance. Collectively, these features position the TME as a critical target for therapeutic intervention, either directly (e.g., anti-angiogenic and stromal-modulating agents) or indirectly by combining ICIs with anti-inflammatory or metabolic modulators that recondition the microenvironment [21,22,23].

### 2.4. Chronic Inflammation, IL-6/PI3K Signaling, and Pharmacological Impact

Tumors of the Head and Neck often arise in the context of chronic inflammation, which can profoundly shape the tumor microenvironment (TME) and influence responsiveness to ICIs. A unifying theme in recent research is that persistent inflammatory stimuli (and the signaling pathways they activate) can lead to an immunosuppressive TME that fosters resistance to immune checkpoint blockade. In particular, the cytokine interleukin-6 (IL-6) and the PI3K/Akt/mTOR signaling pathway have emerged as key links [24,25,26,27]. Several chronic oral conditions illustrate this linkage. Oral lichen planus (OLP) and chronic graft-versus-host disease (cGvHD) of the oral mucosa are inflammatory disorders associated with elevated local IL-6 levels. In OLP, sustained T cell-mediated inflammation causes NF-κB activation and IL-6 production in the epithelium. Indeed, patients with active OLP show higher salivary IL-6, and those with malignant transformation to oral carcinoma exhibit dramatically increased IL-6 [28,29]. How does this relate to immunotherapy resistance? IL-6 and PI3K/Akt/mTOR signaling both promote an immunosuppressive tumor microenvironment that can blunt the efficacy of ICIs. IL-6 is not only a growth factor for epithelia but also a powerful modulator of immune cells. In HNSCC tumors, IL-6 (often produced by tumor or stromal cells under chronic inflammation) drives the polarization of tumor-associated macrophages (TAMs) toward an M2, pro-tumoral phenotype [30,31].

Hyperactivation of the PI3K/Akt/mTOR pathway in tumor cells likewise confers immune resistance. Constitutive PI3K/Akt signaling (often from PTEN tumor suppressor loss or PIK3CA mutations common in HNSCC) enables cancer cells to proliferate and survive under immune attack, and also alters the TME. Notably, PTEN loss in tumor cells causes upregulated immunosuppressive cytokines and chemokines, leading to exclusion of CD8 T cells and enrichment of Tregs in the tumor, ultimately rendering anti–PD-1 therapy ineffective [32,33,34].

Importantly, this model is supported by the overlap of pathways: chronic inflammation → IL-6/NF-κB → PI3K/Akt → epithelial–mesenchymal transition (EMT) and tumor invasion, as well as immune evasion. PI3K/Akt/mTOR is a central hub connecting oncogenic signals (p53 mutation, MAPK activation) with inflammatory signals (NF-κB, IL-6). In HNSCC, the activation of these pathways has been linked to both cancer progression and poor treatment response. Therefore, in patients with chronic inflammatory precursor lesions or high IL-6/PI3K signatures in their tumors, standard immunotherapy may underperform [35,36]. This translational insight suggests that co-targeting the inflammation/PI3K axis could alleviate ICI resistance. By interrupting the IL-6 → STAT3 or PI3K → immunosuppressive circuits, we might convert an immune-cold tumor into an immune-responsive one. Indeed, preclinical data show that inhibiting key nodes (like using IL-6 blockers or PI3K inhibitors) can decrease MDSCs and Tregs and enhance T-cell infiltration, thereby sensitizing tumors to checkpoint blockade.

## 3. The Evolution of Targeted Therapies and Immune Checkpoint

The introduction of immune checkpoint inhibitors (ICIs) has reshaped therapy for Head and Neck squamous cell carcinoma (HNSCC) over the past several years (Table 1). Until the late 2010s, the standard first-line regimen for recurrent or metastatic (R/M) HNSCC was the EXTREME protocol (cetuximab with platinum and 5-FU). The paradigm shifted in 2019 with the phase III KEYNOTE-048 trial, which demonstrated superior survival with the anti–PD-1 antibody pembrolizumab. At approximately 4-year follow-up, pembrolizumab monotherapy improved median overall survival (OS) versus EXTREME in PD-L1-positive tumors (hazard ratio HR 0.61 for PD-L1 combined positive score (CPS) ≥20) and was noninferior in the total population. Pembrolizumab + chemotherapy significantly improved OS in all patients regardless of PD-L1 status (HR 0.71 in all comers). These findings led to regulatory approvals: pembrolizumab became a first-line standard (monotherapy for PD-L1–expressors or with chemo for all patients). This was the first major advance in decades, replacing cetuximab-based therapy. Notably, nivolumab (another anti–PD-1) had already been approved in 2016 for platinum-refractory HNSCC based on CheckMate-141, which showed a survival benefit in second-line treatment. By 2018–2019, ICIs were firmly integrated into clinical practice: pembrolizumab or nivolumab is recommended after platinum failure, and pembrolizumab entered first-line care [37,38,39,40,41].

Since 2018, efforts have extended ICIs into earlier disease settings and combined-modality therapy. Unfortunately, some early trials were negative. The phase III JAVELIN Head and Neck 100 trial tested adding the PD-L1 inhibitor avelumab to standard chemoradiotherapy for locally advanced HNSCC, but found no improvement in progression-free survival (PFS) or overall survival. Similarly, the phase III KEYNOTE-412 trial (pembrolizumab with chemoradiation in locally advanced disease) did not meet its endpoint (reported in 2023) [42,43].

Peri-operative PD-1 blockade is emerging as a promising strategy. KEYNOTE-689 reported a statistically significant event-free survival improvement with neoadjuvant and adjuvant pembrolizumab plus standard surgery/radiotherapy in resectable stage III–IV HNSCC. Neoadjuvant PD-1 may induce tumor regression and prime systemic immunity; early-phase studies (e.g., IMCISION) reported major pathological responses, although survival benefit remains to be established. If confirmed by guideline updates and regulatory decisions, this approach could reshape curative-intent algorithms for biologically selected patients [44,45,46].

PD-L1 CPS remains the most practical predictor in HNSCC. Tumor HPV status, TMB, and interferon-γ-related signatures offer complementary prognostic information, whereas PI3K activation/IL-6 and myeloid-dominant TMEs flag potential resistance, supporting rational ICI combinations. Furthermore, there has been intense interest in combining ICIs to amplify anti-tumor immunity. The CTLA-4 inhibitor ipilimumab has been paired with PD-1 blockade in other cancers (e.g., melanoma) and tested in HNSCC. The phase III CheckMate-651 trial (first-line nivolumab + ipilimumab vs. EXTREME chemotherapy) showed a positive OS trend but did not reach statistical significance, even in PD-L1 high subsets. Final results confirmed no significant OS benefit for nivo + ipi over standard chemo (median OS ~13.9 vs. 13.5 months). Despite the lack of approval from that trial, some patients achieved long-term disease control on dual-checkpoint blockade. A case report even documented a refractory HNSCC patient salvaged by nivolumab + ipilimumab after failing other treatments. Beyond CTLA-4, next-generation checkpoints are being actively investigated. LAG-3 is a T-cell inhibitory receptor upregulated during PD-1 therapy; relatlimab (anti-LAG-3) combined with nivolumab showed success in melanoma and is now in HNSCC trials. For example, the TACTI-002 study combined eftilagimod (a soluble LAG-3 Ig fusion) with pembrolizumab in metastatic HNSCC and reported encouraging response rates. Clinical trials of nivolumab + relatlimab (NCT04080804) and nivolumab + ipilimumab (NCT04326257) in HNSCC are ongoing to assess activity [47,48,49,50,51].

Another target is TIGIT, an inhibitory receptor on T cells and NK cells. Several phase I–II trials are combining anti-TIGIT antibodies with PD-1/PD-L1 blockade in HNSCC. For instance, tiragolumab (anti-TIGIT) plus atezolizumab is being tested as neoadjuvant therapy in resectable HNSCC. Other novel checkpoints under exploration include TIM-3, CD96, and others, each aiming to overcome the “adaptive resistance”, whereby tumors upregulate alternate immunosuppressive pathways under PD-1 blockade. While none of these combinations have yet earned regulatory approval in Head and Neck cancer as of 2025, they represent anticipated breakthroughs. Early-phase results suggest that dual-checkpoint inhibition can deepen response in a subset of patients, and the field awaits mature survival data [52,53,54,55].

The variable efficacy of ICIs in HNSCC (overall response rates ~15–20% in unselected patients) underscores the importance of biomarkers. PD-L1 expression by immunohistochemistry is the most established predictor of benefit. Trials stratified outcomes by combined positive score (CPS), which counts PD-L1 on tumor and immune cells. In KEYNOTE-048, patients with high PD-L1 (CPS ≥ 20) derived the greatest benefit from pembrolizumab monotherapy (median OS 14.9 vs. 10.7 months vs. chemo). Those with CPS < 1 had little benefit from monotherapy, so these patients usually require combination with chemotherapy. PD-L1 CPS now guides first-line therapy choices.[56] Beyond PD-L1, tumor HPV status is a unique factor in Head/Neck cancers. Virus-positive oropharyngeal cancers may be more immunogenic; however, immunotherapy trials have shown mixed results regarding HPV. Some analysts suggest HPV-positive tumors (which often have an inflamed microenvironment) respond somewhat better, but differences are not dramatic.[57,58] Tumor mutational burden (TMB) is another biomarker of interest: a small fraction of HNSCC has high TMB, and these may respond well to PD-1 blockade (pembrolizumab has tissue-agnostic approval for TMB ≥ 10).[59] Gene expression profiles indicating T-cell inflammation (interferon-γ signature) have also correlated with response in exploratory analyses. On the other hand, immunosuppressive features like high neutrophil-to-lymphocyte ratio (NLR) in blood or an abundance of M2 macrophages in tumors, correlate with poor outcomes. As for predictive biomarkers of resistance, emerging data highlight factors such as PTEN loss or PI3K pathway activation in tumors (which can exclude T cells) and certain cytokines (elevated IL-6 or IL-8), as markers of an immune-cold tumor. Ongoing trials are incorporating biomarker-driven arms, for example, some investigate ICI combinations in patients selected for specific checkpoint ligand expression (e.g., TIGIT expression for anti-TIGIT therapy). Overall, while PD-L1 remains the key clinical biomarker, research from 2018 to 2025 has broadened our understanding that HNSCC response to immunotherapy depends on a complex interplay of viral status, mutational landscape, and the tumor immune contexture [60,61,62,63,64].

### 3.1. Drugs That Target the Epidermal Growth Factor Receptor

The human epidermal growth factor receptor tyrosine kinase family includes the cytoplasmic transmembrane protein known as epidermal growth factor receptor (EGFR). It typically consists of intracellular domains with tyrosinase kinase activity, transmembrane domains, and extracellular ligand-binding domains [65]. Neuregulin, epiregulin, and transforming growth factor-α (TGF-α) are examples of endogenous ligands. These endogenous ligands can form homologous or heterogeneous dimers when they bind to the extracellular domain of EGFR. These dimers activate the tyrosine kinases, leading to autophosphorylation of tyrosine residues and triggering several downstream signaling pathways, including the Ras-Raf-mitogen-activated protein kinase pathway and the phosphatidylinositol 3-kinase/protein kinase B (PI3K/Akt) pathway, which resulted in antiapoptosis, tumor cell proliferation, metastasis, and angiogenesis [66,67].

Currently, two different kinds of medications are employed in contrast to this target. These medications include tyrosine kinase inhibitors (TKIs) such as erlotinib, gefitinib, and afatinib, and monoclonal antibodies, such as cetuximab and nimotuzumab. By attaching to the extracellular domain of EGFR, the monoclonal antibodies block the connection between ligands and prevent signals from entering the cell [68]. Cetuximab, an immunoglobulin G1 (IgG1) monoclonal antibody, is used in combination with radiation therapy as first-line of treatment for advanced Head and Neck squamous cell carcinoma. By attaching to the extracellular ligand-binding region of EGFR, cetuximab effectively blocks endogenous ligand-activated receptors, leading to a decrease in angiogenesis, invasion, metastasis, and proliferation while promoting cell death [69]. For platinum-resistant recurrent or metastatic Head and Neck squamous cell carcinomas, cetuximab monotherapy exhibits a response rate of 10–13% and a PFS of 2.2–2.8 months [70]. Nimotuzumab, a humanized IgG1 monoclonal antibody, is used to treat glioblastoma, nasopharyngeal cancer, and Head and Neck squamous cell carcinoma. Its lengthy half-life and moderate affinity, when compared to cetuximab, significantly reduce side effects, like skin toxicity and immunogenicity. By inhibiting the survival, growth, and angiogenesis of cancer cells, nimotuzumab directly contributes to anticancer effects [71,72].

### 3.2. Drugs That Target the Programmed Cell Death Receptor

PD-1, or programmed cell death receptor-1, is linked to the CD28 family. Natural killer cells, T cells, B cells, dendritic cells, and macrophages all express PD1. Tumor immune escape occurs when PD1 attaches to PD-L1 (programmed cell death ligand 1), causing effector T cells to undergo apoptosis. Additionally, it promotes the production of interleukin-10 cytokines, which reduce inflammation. According to a number of studies, 50–90% of OSCC patients have overexpressed PD-L1. The metastasis of cervical lymph nodes is positively connected with this elevated expression [73,74,75].

The prognosis of many malignant tumors, including OSCC and melanoma, was comparable to their co-expressions. Two medications are currently utilized to target PD-1: pembrolizumab and nivolumab. The U.S. Food and Drug Administration has approved the use of these medications for the treatment of advanced melanoma. Patients with Head and Neck squamous cell carcinoma are treated with pembrolizumab [76].

### 3.3. Drugs That Target Inhibitors of Cyclin-Dependent Kinase (CDK)

The excessive growth of malignant cells is linked to changes in the expression of cyclin-dependent kinases (CDKs). Cyclin and its regulatory partner, CDKs, often control the cell cycle. Cell cycle CDKs and transcriptional CDKs are the two subgroups into which these CDKs are separated. The excessive growth of cancerous cells is linked to the changed expression of CDKs. Typically, cyclin and its regulatory partner, CDKs, control the cell cycle. Cell cycle CDKs and transcriptional CDKs are the two subgroups into which these CDKs are classified [77].

Chang et al. reported that OSCC had 17.2-times the expression of the CDK1 gene compared to normal tissue. This overexpression is associated with malignant behaviors.[78] Chen et al. also found that the CDK1 protein is overexpressed in patients with lymph node metastases or recurrent OSCC [79]. Thus, CDK1 expression can be an OSCC survival prognostic biomarker. Because CDKs are important for cellular transcription and cell cycle control, they become obvious targets for anticancer treatment. Numerous studies show that CDK inhibitors may be used therapeutically to treat a range of illnesses, including diabetes, cancer, infectious disorders, and renal diseases [80].

### 3.4. Drugs That Target the Receptor Inhibitors of the Vascular Endothelial Growth Factor

Tumor growth and metastasis are significantly influenced by tumor angiogenesis. Therefore, it is recommended that angiogenesis be suppressed in order to treat OSCC effectively. Vascular endothelial growth factor (VEGF) is a mitogen unique to endothelial cells and referred to as an angiogenic factor. OSCC has high expression of VEGF, a chemical crucial for tumor angiogenesis. Monoclonal antibodies like bevacizumab and multikinase inhibitors like vandetanib and sorafenib are examples of substances that work against VEGF and its receptors [81,82]. A humanized monoclonal antibody called bevacizumab targets VEGF-A. By lowering intra-tumor pressure and microvascular permeability, it suppresses angiogenesis and improves the distribution of chemotherapeutic medicines to tumor cells [83].

Sorafenib is a multitargeted and multikinase inhibitor that inhibits the growth and proliferation of tumor cells, as well as tumor angiogenesis by blocking its various targets, including Raf serine/threonine kinase, c-Kit, platelet-derived growth factor receptor β (PDGFR-β), and VEGFR (vascular endothelial growth factor receptor) 1–3. The downregulation of Mcl-1 triggers tumor cell death. Radiation and sorafenib are combined to reduce nuclear factor kappa B activity, which has a synergistic effect on OSCC cells [84].

The VEGFR-2 and EGFR tyrosine kinase activities are sufficiently inhibited by the tyrosine kinase receptor vannetanib. According to the findings of preclinical research, vandetanib suppresses the growth of OSCC and other xenograft tumor cells. Vandetanib may be able to overcome EGFR inhibitor resistance in preclinical trials when combined with cisplatin and radiation therapy [85].

### 3.5. Drugs That Target the Mammalian Target of Rapamycin Inhibitors

Mammalian target of rapamycin (mTOR) is a serine/threonine protein kinase. It regulates proliferation, the cell cycle, and cell survival. The regulation of cell growth and proliferation is significantly influenced by the PI3K/AKT signal pathway. Angiogenesis, invasion, metastasis, and tumor formation are all primarily influenced by mTOR, a downstream molecule of the PI3K/AKT signal pathway [86]. According to a study by Liao et al., 85 out of 160 patients with tongue squamous cell carcinoma had overexpressed phosphorylated mTOR [87].

First-generation and second-generation mTOR inhibitors are the two sub-types of mTOR inhibitors. Rapamycin was the basis for the development of first-generation inhibitors. Rapamycin and cytoplasmic protein, namely peptidyl-prolyl cis-trans isomerase tacrolimus-binding protein, combine to form a complex. Everolimus and temsirolimus are analogs of rapamycin. Second-generation mTOR inhibitors are PP242, Torin 1, and PP30 [88,89].

Temsirolimus, an intravenous drug, is used to treat kidney cancer. Numerous investigations and clinical trials demonstrate that temsirolimus inhibits the growth of squamous cell carcinoma of the Head and Neck. Rapamycin derivative, everolimus, is used to treat kidney cancer by suppressing the immune system. Everolimus has anti-angiogenesis and anticancer effects in the treatment of Head and Neck squamous cell carcinoma, according to numerous studies [90,91,92].

## 4. Drug Repurposing Opportunities: Targeting Inflammation and PI3K/MTOR to Enhance Immunotherapy

Chronic inflammatory signaling (e.g., IL-6/STAT3) and PI3K/AKT/mTOR activation contribute to an immune-excluded, myeloid-dominant tumor microenvironment (TME) and primary resistance to PD-1. Repurposed agents that modulate these nodes, often inexpensive and well characterized, may recondition the TME, increase T-cell infiltration/function, and augment ICI efficacy. Below, we map candidate drugs to their mechanistic interception points and evidence tier (preclinical, observational/retrospective, early prospective), with pragmatic safety notes [93,94].

Metformin, a widely used antidiabetic agent, has shown immunomodulatory and anti-inflammatory effects in cancer settings. Mechanistically, metformin activates AMPK and indirectly inhibits mTORC1, counteracting hyperactive PI3K/AKT/mTOR signaling often seen in inflamed or PTEN-deficient tumors (Table 2). Within the tumor stroma, metformin can dampen pro-inflammatory cytokine production; for example, in vitro studies show inhibition of NF-κB in cancer-associated fibroblasts, reducing IL-6 secretion. By lowering IL-6 and related factors, metformin may mitigate M2 macrophage polarization and T-cell suppression driven by chronic inflammation. Metformin has also been reported to down-regulate PD-L1 on tumor cells via the IL-6/JAK2/STAT3 pathway. Furthermore, metformin improves metabolic conditions in the TME, thus normalizing tumor vasculature and reducing hypoxia, which may help rescue exhausted T cells. Clinically, observational analyses suggest improved ICI responses in diabetic cancer patients on metformin in some cohorts. Metformin can also increase short-chain fatty acids (SCFAs) through microbiome modulation, contributing to histone deacetylase (HDAC) inhibition and dampening tumor-promoting inflammation. In oral cancer, metformin appears to act through organic cation transporters (OCT3), which are predominantly elevated in both OPMDs and OSCCs [95,96,97,98,99,100,101,102].

Statins (HMG-CoA reductase inhibitors): Statins (e.g., atorvastatin, simvastatin) are cholesterol-lowering drugs with notable anti-inflammatory and immunomodulatory activities. They inhibit the mevalonate pathway, reducing cholesterol synthesis and impairing prenylation of signaling proteins such as RAS, with downstream effects on PI3K/AKT and other survival pathways. Preclinical and clinical observations suggest statins may favor a more T-cell permissive TME, potentially enhancing ICI activity in HNSCC. Clinical studies report associations between statin use and improved ICI outcomes, potentially via PD-L1 modulation and effects on myeloid cells. Statins may also reduce endothelial dysfunction, thus mitigating radiation-induced vascular injury. Pravastatin has been associated with reduced radiation-induced fibrosis (RIF), while one cohort reported a 53% lower incidence of hearing loss with atorvastatin. These signals warrant prospective confirmation and careful attention to drug–drug interactions (e.g., CYP3A4) and myopathy risk during CR [103,104,105,106,107].

Celecoxib and NSAIDs (COX-2 inhibition): Chronic inflammation in tumors is often driven by the COX-2/prostaglandin-E2 pathway. COX-2 is frequently upregulated in HNSCC and in some premalignant lesions, leading to elevated PGE2. PGE2 recruits MDSCs and IL-10-producing macrophages and impairs dendritic cell function. COX-2 inhibitors such as celecoxib reduce MDSC accumulation and enhance T-cell infiltration in preclinical models. Clinical observations—particularly in biologically defined subsets—suggest NSAIDs may improve immunotherapy outcomes, although prospective HNSCC data remain limited. Given accessibility and mechanistic plausibility, COX-2 inhibition is a rational adjunct under trial conditions, with peri-operative bleeding and gastrointestinal risks carefully managed [108,109,110,111].

Hydroxychloroquine (autophagy inhibition and microenvironmental effects): Hydroxychloroquine (HCQ), an antimalarial and immunomodulatory drug, has gained interest in oncology as an autophagy inhibitor and modifier of the TME. HCQ blocks autophagy in tumor cells, enhancing susceptibility to T-cell-mediated killing. It can repolarize macrophages from an M2 to an M1 phenotype, decrease MDSCs and Tregs, and improve dendritic cell function. Preclinical HNSCC models show HCQ can synergize with anti–PD-1 therapy to improve tumor control. Early-phase clinical evaluation is ongoing in solid tumors; HNSCC-specific trials are needed to define benefit–risk and dosing windows (with ocular and QT monitoring) [112,113,114,115].

Mitochondrial/metabolic disruptors (menadione, pyrvinium pamoate, atovaquone, antimycin A). These agents target oxidative phosphorylation and tumor bioenergetics to induce metabolic stress, potentially exposing tumors to immune attack and limiting stemness/EMT. Evidence in OSCC/OPSCC is preclinical; clinical use should be confined to trials due to off-target toxicities and uncertain therapeutic windows [116,117,118].

Propranolol (β-adrenergic antagonism). Propranolol, a non-selective β-blocker, may enhance the action of 5-FU and cisplatin in part via increased PTEN expression and attenuation of pro-survival signaling. While mechanistic and early clinical signals are encouraging, robust HNSCC data are limited and prospective trials are required [119,120,121].

In summary, the inflammation–PI3K–immunity framework delineates multiple points for therapeutic interception with existing drugs. Agents such as metformin, statins, COX-2 inhibitors, and hydroxychloroquine target distinct nodes in this network, from lowering IL-6 and PD-L1 to reprogramming myeloid populations and constraining tumor survival pathways. These strategies are hypothesis-driven and remain investigational in HNSCC; by repurposing them rationally, ideally in biomarker-selected settings and within peri-operative or post-CRT windows, we may extend the benefits of immune checkpoint blockade to more patients. Prospective, biomarker-guided trials will be essential to define efficacy, safety, and optimal combinations.

## 5. Expected Breakthroughs

Over the near term, peri-operative PD-1 strategies and biomarker-driven dual-checkpoint approaches are the most credible candidates to modify care pathways in OSCC/OPSCC. The positive KEYNOTE-689 perio-perative immunotherapy results are expected to change practice, bringing ICIs into the curative setting. This could improve cure rates for locally advanced oral cancers by eradicating microscopic disease. Novel ICIs targeting checkpoints beyond PD-1 (such as LAG-3 or TIGIT) may soon yield phase III data; a successful outcome could lead to the first dual-checkpoint regimen approved in HNSCC. Another exciting avenue is immunoprevention—using immunotherapy in premalignant lesions to prevent cancer development. Chronic oral premalignant conditions sometimes progress to carcinoma via immune evasion. A recent phase II trial treating high-risk oral leukoplakia with nivolumab showed a 2-year cancer-free survival of 73%, suggesting that ICIs might intercept malignant transformation in the oral cavity. Moreover, therapeutic vaccines (e.g., HPV-targeted vaccines for HPV-positive HNSCC) combined with ICIs are being explored to further boost anti-tumor immunity. Finally, efforts to personalize immunotherapy dosing and sequence (e.g., induction ICI before standard therapy, or adjuvant ICI guided by minimal residual disease biomarkers) are ongoing. In summary, from 2018 to 2025, ICIs moved from a last-resort palliative therapy to a central component of HNSCC management, and the coming years promise even broader integration—tailoring immunotherapy by tumor biology, combining checkpoints, and extending benefits to more patients in both metastatic and curative settings.

Latest research is discovering innovative approaches, and nanomaterials are being leveraged as vehicles for transporting anticancer drugs. CpG, a Toll-like receptor 9 (TLR-9) agonist, is loaded onto molybdenum disulfide nanosheets, activating dendritic cells. Self-assembly protein nano-carriers are being used to target CXCR4-positive cancers. Chitosan Sponge Matrix formed by the assembly of Chitosan nanoparticles are able to carry cisplatin, enhancing its efficacy. Smart drug delivery systems combine nanotechnology with targeting strategies like drug release based on pH, temperature, or biochemical changes. One system uses pH-functionalized, charge-adjustable nanoparticle delivering oxaliplatin and miRNA [122,123,124,125]. Extracellular vesicles (EVs) with low immunogenicity and excellent biocompatibility are ideal for drug delivery. They can be used to transport chemotherapeutic agents, RNAs, small molecules, and carry gene therapies [126,127,128]. Integration of pharmacology, bioinformatics, and network analysis, focusing on comprehensive mapping of genes involved in oral and oropharyngeal cancer and drugs has led to an innovative branch of network pharmacology. This method systematically analyses mechanisms, potential targets, and novel therapeutic interventions, leading to rapid drug discoveries. Translation into practice will depend on confirmatory outcomes, safety in real-world multimodal care, and alignment with guideline adoption.

## 6. Conclusions

Progress in comprehending oral cancer pathophysiology, along with the innovation of novel diagnostic and therapeutic strategies, may substantially enhance patient outcomes. The incorporation of molecular biology into clinical practice has resulted in the advancement of targeted medicines and immunotherapies, providing more individualized therapy alternatives. Ongoing investigation into the molecular determinants of oral cancer, together with the formulation of innovative therapeutic approaches, is crucial for enhancing survival rates and the quality of life for individuals afflicted by this debilitating illness.

As a narrative (non-systematic) review, our selection may be prone to publication and selection bias. HNSCC subsites and HPV status introduce biological heterogeneity that can dilute generalizability. Signals for repurposed agents are largely preclinical or observational; prospective, biomarker-guided studies, particularly in the peri-operative setting, remain mandatory before routine adoption.

## Figures and Tables

**Table 1:** Various molecular target pathways, mechanism of action, and representative drugs.

Target/Pathway	Mechanism of Action	Drugs
EGFR (Epidermal Growth Factor Receptor)	Tyrosine kinase inhibitors (TKIs) bind to the intracellular ATP-binding site (reversible or irreversible) → block kinase autophosphorylation and downstream signaling Monoclonal antibodies (mAbs) bind to the extracellular ligand-binding domain, preventing ligand binding/receptor dimerization/receptor activation	TKIs: gefitinib, erlotinib, afatinib, dacomitinib, osimertinib, mobocertinib, icotinib, and sunvozertinib mAbs/biologics: cetuximab, necitumumab, panitumumab, nimotuzumab, and amivantamab (bispecific EGFR/MET)
PD-1/PD-L1 (Immune Checkpoint)	Monoclonal antibodies block the interaction between PD-1 (on T cells) and PD-L1 (on tumor cells or antigen-presenting cells), preventing the inhibitory signal that dampens T-cell activation/enabling T-cell-mediated tumor killing	Anti–PD-1: pembrolizumab, nivolumab, cemiplimab, and dostarlimab Anti–PD-L1: atezolizumab, durvalumab, and avelumab
CDKs (Cyclin-Dependent Kinases)	Small molecule inhibitors of CDKs inhibit kinase activity and block phosphorylation of downstream effectors (e.g., Rb) → cell cycle arrest, especially in G1/S transition	Palbociclib, ribociclib, and abemaciclib (CDK4/6 inhibitors)
VEGF/VEGFR (Angiogenesis Pathway)	Monoclonal antibodies bind VEGF ligands → sequester them and receptor tyrosine kinase inhibitors (RTKIs) inhibit VEGFR kinase activity (or broader multikinase inhibition)	Bevacizumab (anti-VEGF) and ziv-aflibercept; RTKIs/multikinase inhibitors: sunitinib, sorafenib, pazopanib, axitinib, lenvatinib, and vandetanib
mTOR (Mammalian Target of Rapamycin)	Small-molecule inhibitors of mTOR block the kinase activity (TORC1 or both TORC1/TORC2) → suppress cell growth, protein synthesis, and metabolism	Rapamycin (sirolimus), everolimus, and temsirolimus; second-generation TOR inhibitors (e.g., PP242, Torin1)

**Table 2:** Various drugs repurposed as anticancer agents, their mechanism of action, and clinical/preclinical evidence.

Interception Mode/Pathway	Repurposed Agent	Mechanism of Action	Evidence (in Cancer with Immunotherapy Context)	Safety/Practical Notes
AMPK activation → indirect mTORC1 inhibition/metabolic reprogramming/anti-inflammation	Metformin	Activates AMPK → suppression of mTORC1. Lowers systemic insulin/IGF signaling Reduces NF-κB/IL-6/STAT3 signaling in stroma/CAFs. Down-regulates PD-L1 expression (via IL-6/JAK2/STAT3). Improves metabolic fitness/reduces hypoxia in TME.	Observational/retrospective: Diabetic patients on metformin showed improved responses to ICI in some cohorts. Metformin + anti–PD-1 synergy in murine models. Early prospective/mechanistic: Increasing interest, e.g., metformin co-treatment “boosts immunotherapy” reviews.	Metformin is generally well tolerated, with long use in diabetes. Risk of lactic acidosis in renal impairment or hypoxia.
Mevalonate/prenylation/RAS/cholesterol/immunomodulation	Statins (e.g., atorvastatin, simvastatin)	Inhibit HMG-CoA reductase → reduce cholesterol, isoprenoid pathway. Impair prenylation of small GTPases (e.g., RAS/Rho) → downstream PI3K/AKT signaling interference. Anti-inflammatory effects: reduce cytokine secretion, modulate myeloid cell polarization, lower endothelial activation. May modulate PD-L1, myeloid suppression.	Preclinical/mechanistic: Statins shown to enhance chemo/overcome resistance; immunomodulatory signals in tumor models. Observational/retrospective: Some cancer immunotherapy cohorts report improved outcomes in statin users (various cancers, though not robust HNSCC--specific). Early prospective: Few trials explicitly combining statins + ICI	Main risks: myopathy, hepatic effects, drug–drug interactions (notably via CYP3A4). Careful monitoring during CRT (e.g., overlapping toxicity). Need attention to lipophilicity vs. hydrophilicity (some statins penetrate tissues better).
COX-2/prostaglandin E_2_ axis/immunosuppressive inflammation	Celecoxib (selective COX-2 inhibitor), NSAIDs	Inhibit COX-2 → reduce PGE_2_ production. Lower recruitment/activation of MDSCs, suppress IL-10 macrophages and improve DC and T-cell infiltration. Downstream reduction in immunosuppressive milieu.	Preclinical: Many tumor models show COX-2 inhibition enhances T-cell infiltration and reduces immune suppression. Observational/retrospective: Some ICI cohorts show NSAID/COX-2 use correlates with better outcomes (heterogeneous). Early prospective: Limited, especially in HNSCC.	COX-2 inhibitors carry GI bleeding risk and cardiovascular risk. In peri-operative/CRT settings, bleeding risk must be managed. Dose, timing, and patient selection are critical to mitigate toxicity.
Autophagy/lysosome inhibition/microenvironment remodeling	Hydroxychloroquine (HCQ)/Chloroquine (CQ)	Blocking autophagic flux in cancer cells, increasing tumor susceptibility to T-cell killing. Repolarizing macrophages from M2 → M1, reducing MDSCs and regulatory T cells, improving DC function. Modulation of tumor stroma and vasculature, CAFs via lysosomal/TLR/NF-κB mechanisms.	Preclinical: Strong support in many tumor models, HCQ/CQ + immunotherapy synergy in animal models. Clinical/early trials: Multiple cancer trials (various tumor types) with HCQ combinations ongoing. Some early signals in solid tumor trials combining HCQ/CQ + chemotherapy/targeted therapy (phase I)	Retinopathy/ocular toxicity risk with long-term use. QT prolongation risk and GI side effects Dosing needs careful calibration (in many trials, MTD or tolerability is limiting). The degree of autophagy dependence varies by tumor and context.
Bioenergetic/mitochondrial metabolism disruptors	Menadione (vitamin K3), pyrvinium pamoate, atovaquone, antimycin A (experimental)	Disrupt oxidative phosphorylation/mitochondrial electron transport → metabolic stress in tumor cells. Collapse of tumor metabolic plasticity → increase immunogenicity, weaken stemness/EMT.	Preclinical: Evidence in cell/animal models of metabolic stress + immunotherapy synergy (largely non-HNSCC). Currently little clinical data in humans for cancer immunotherapy settings.	High risk of off-target toxicities (mitochondrial toxicity). Narrow therapeutic windows; careful monitoring required. Likely restricted to experimental/trial settings.
β-adrenergic/stress signaling modulation	Propranolol (and possibly other β-blockers)	Block β-adrenergic signaling → increase PTEN expression and reduce pro-survival signaling. Modulate stress-induced immune suppression and reduce catecholamine-driven immunosuppression.	Preclinical/mechanistic: some tumor and chemo combination models show enhanced sensitivity. Observational/retrospective: In various cancers, β-blocker use has been associated with improved survival/reduced metastasis. Prospective in immunotherapy combinations: Very limited in HNSCC.	β-blockers have known cardiopulmonary side effects (bradycardia, hypotension, and bronchospasm). Must be used with caution in patients with comorbidities. Dose, timing, and cancer specificity are uncertain.

## Abbreviations

The following abbreviations are used in this manuscript:

CAFs	Cancer-Associated Fibroblasts
CDK	Cyclin-Dependent Kinase
cGvHD	Chronic Graft-Versus-Host Disease
CPS	Combined Positive Score
ECM	Extracellular Matrix
EGFR	Epidermal Growth Factor Receptor
EMT	Epithelial–Mesenchymal Transition
EVs	Extracellular Vesicles
HCQ	Hydroxychloroquine
HDAC	Histone Deacetylase
HNCs	Head and Neck Cancers
HNSCC	Head and Neck Squamous Cell Carcinoma
ICIs	Immune Checkpoint Inhibitors
IgG1	Immunoglobulin G1
IL-6	Interleukin–6
MMPs	Matrix Metalloproteinases
mTOR	Mammalian Target of Rapamycin
NSAIDs	Nonsteroidal Anti-Inflammatory Drugs
OCT3	Organic Cation Transporters
OLP	Oral Lichen Planus
OPSCC	Oropharyngeal Squamous Cell Carcinoma
OSCC	Oral Squamous Cell Carcinoma
P13K/Akt	Phosphatidylinositol 3-Kinase/Protein Kinase B
PD-1	Programmed Cell Death Protein 1
PD-L1	Programmed Cell Death Ligand 1
SCFAs	Short-Chain Fatty Acids
TAMs	Tumor-Associated Macrophages
TGF-α	Transforming Growth Factor–α
TLR-9	Toll-like Receptor-9
TMB	Tumor Mutational Burden
TME	Tumor Microenvironment
VEGF	Vascular Endothelial Growth Factor
